# Associations between physical activity and ankle-brachial index: the Swedish CArdioPulmonary bioImage Study (SCAPIS)

**DOI:** 10.1186/s12872-024-04137-x

**Published:** 2024-08-28

**Authors:** Ensieh Memarian, Viktor Hamrefors, Isabella Kharraziha, Göran Bergström, Anders Blomberg, Andrei Malinovschi, Carl Johan Östgren, Örjan Ekblom, Gunnar Engström, Anders Gottsäter

**Affiliations:** 1 Department of Clinical Sciences in Malmö, Department of Internal Medicine, Lund University, Skåne University Hospital, Jan Waldenströms gata 15, 5th floor, Malmö, S-20,502 Sweden; 2 Department of Cardiology, Skåne University Hospital, Malmö, Sweden; 3 Department of Molecular and Clinical Medicine, Institute of Medicine, Sahlgrenska Academy, University of Gothenburg, Gothenburg, Sweden; 4 Department of Clinical Physiology, Sahlgrenska University Hospital, Region Västra Götaland, Gothenburg, Sweden; 5 Department of Public Health and Clinical Medicine, Umeå University, Umeå, Sweden; 6 Department of Medical Sciences, Clinical Physiology, Uppsala University, Uppsala, Sweden; 7 Department of Health, Medicine and Caring Sciences, Linköping University, Linköping, Sweden; 8 Centre for Medical Image Science and Visualization (CMIV), Linköping University, Linköping, Sweden; 9 Department of Physical Activity and Health, The Swedish School of Sport and Health Sciences, Stockholm, Sweden; 10 Department of Neurobiology, Care Sciences and Society, Division of Nursing, Karolinska Institute, Huddinge, Sweden

**Keywords:** ABI, Accelerometer, Physical activity, Sedentary time, CVD

## Abstract

**Background:**

The ankle–brachial index (ABI) is the ratio of the ankle and brachial systolic blood pressures. In the clinical setting, low ABI (< 0.9) is an indicator of peripheral atherosclerosis, while high ABI (> 1.4) is a sign of arterial stiffness and calcification. The purpose of the current study was to investigate the association between ABI and physical activity levels, measured by accelerometer.

**Methods:**

The Swedish CArdioPulmonary bioImage Study (SCAPIS) is a Swedish nationwide population-based cross-sectional cohort for the study of cardiovascular and pulmonary diseases, in which individuals aged 50–64 years were randomly invited from the general population. The study population with data on ABI, physical activity, and sedentary time based on accelerometry was 27,737. Differences between ABI categories and associations to sedentary behavior, moderate to vigorous physical activity (MVPA), and other metabolic characteristics were compared. ABI was categorized as low, ABI ≤ 0.9, borderline, ABI 0.91–0.99, normal, ABI 1.0-1.39, and high, ABI ≥ 1.4.

**Results:**

Prevalence of low ABI was higher in the most sedentary quartiles compared to the least sedentary (0.6% vs. 0.1%, *p* < 0.001). The most sedentary individuals also exhibited higher BMI, higher prevalence of diabetes and hypertension. The proportion of wake time spent in MVPA was lowest in those with low ABI (0.033 ± 0.004; *p* < 0.001) and highest in those with ABI > 1.4 (0.069 ± 0.001; *p* < 0.001) compared to those with normal ABI. Compared to normal ABI, the proportion of sedentary time was highest in those with low ABI (0.597 ± 0.012; *p* < 0.001) and lowest in those with ABI > 1.4 (0.534 ± 0.002; *p* = 0.004).

**Conclusion:**

This population-based study shows that middle-aged individuals with ABI > 1.4 have the highest level of physical activity, while individuals with a lower ABI, especially those with ABI < 0.9, are less active and spend more time sedentary. Future studies are needed to understand the relationships between ABI, physical activity, and the risk of peripheral arterial and cardiovascular disease in the general population.

**Supplementary Information:**

The online version contains supplementary material available at 10.1186/s12872-024-04137-x.

## Background

The ankle–brachial index (ABI), is the ratio of the ankle and brachial systolic blood pressures. ABI is used in clinical settings to diagnose and assess the severity of peripheral artery disease (PAD) in the lower extremities [1, 2]. A low ankle–brachial index (ABI) is also an indicator of generalized atherosclerosis and a subclinical measure of cardiovascular disease (CVD), associated with an increased risk of future cardiovascular events [1, 3–6]. The major risk factors for CVD and PAD are smoking, diabetes mellitus, arterial hypertension, hyperlipidemia, obesity, and a sedentary lifestyle [7–9]. Physical activity is positively associated with ABI in individuals without PAD, and this association remains significant after adjustment for hypertension, smoking, and BMI [10].

Furthermore, both cross-sectional and intervention studies show that even modestly higher levels of physical activity may be beneficial for secondary risk prevention in individuals with low ABI and risk for CVD [11, 12].

Previous studies [11, 12] of physical activity concerning ABI have mainly investigated smaller samples of patients or healthy subjects, resulting in limited statistical power. Additionally, most of the earlier studies had access only to self-reported data on physical activity rather than objective accelerometer-based measures. The purpose of the current study was to investigate the association between ABI and physical activity levels, measured by accelerometer, in a large cohort of middle-aged men and women from the general population.

## Materials and methods

### Study population

The Swedish CArdio Pulmonary bioImage Study, SCAPIS, is a nationwide population-based cohort for the study of CVD and chronic obstructive pulmonary disease (COPD). SCAPIS is a collaboration between six Swedish universities and university hospitals [13]. Subjects aged 50–64 years were randomly selected from the general population and invited by letter. The participation rate was 50%. The exclusion criteria were being unable to either understand the instructions in Swedish language or to complete the questionnaires. The examinations were performed at the screening centers on two or three different days, 1–2 weeks apart during 2013–2018. A total of 30,154 men and women were included in the study. On the first screening day, participants filled in a detailed questionnaire about their lifestyle and living conditions and received an accelerometer to wear for seven days to monitor their daily physical activity (PA). Individuals with < 4 days of accelerometry were excluded. As compared to the final study population, those with < 4 days of registration were more often male (57 vs. 47%), had higher BMI (28.1 vs. 26.9 kg/m^2^), lower mean ABI (1.22 vs. 1.23), higher DBP (78.3 vs. 77.5 mmHg), and had higher prevalence of diabetes (7.4 vs. 4.6%) and smoking (20.1 vs. 12.2%).

**Fig. 1:**
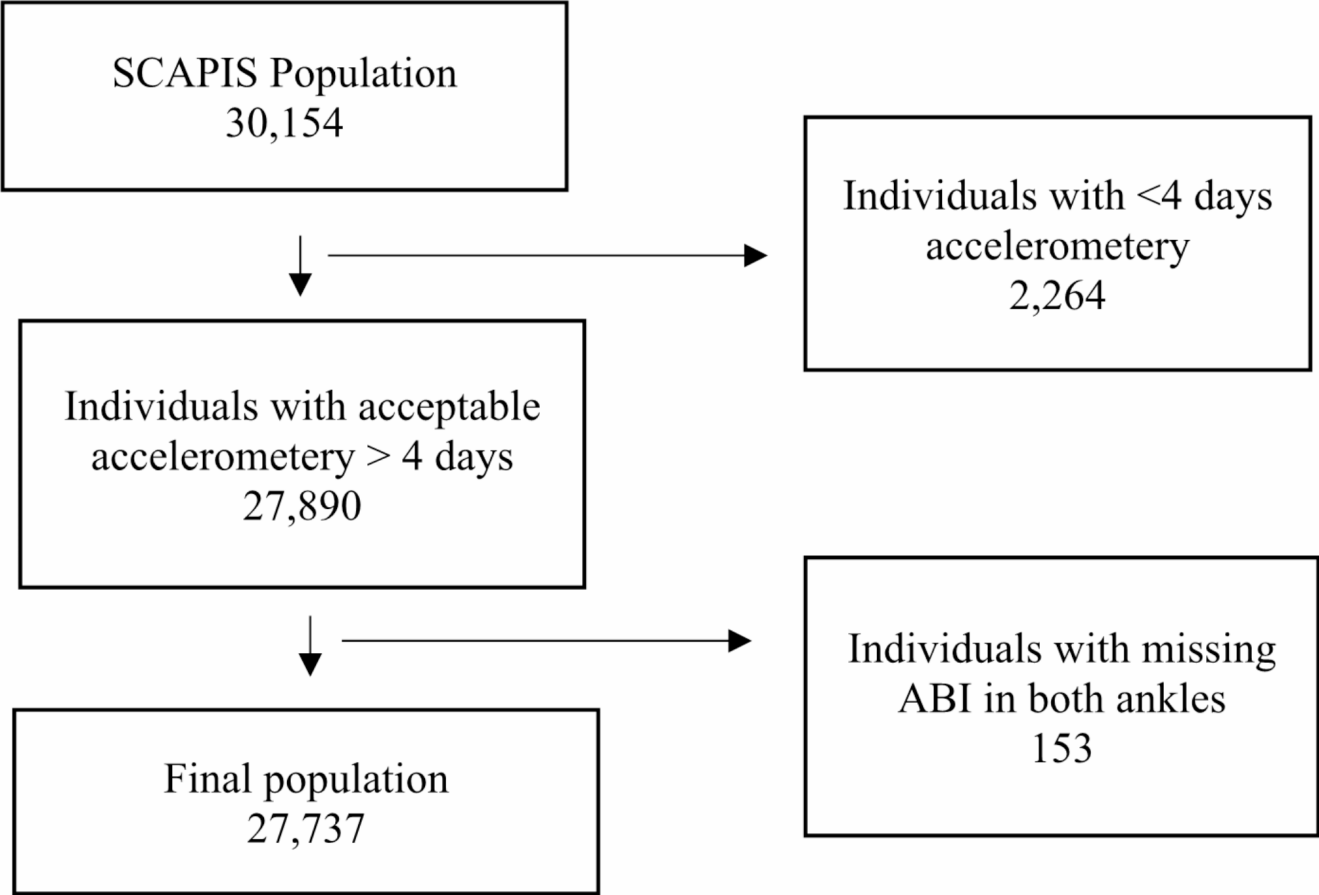
Study population and excluded individuals

Subjects missing ABI measurements in both ankles were excluded. There were 47 subjects with missing ABI in either the left or right ankle (ABI-right = 30, ABI-left = 17), which were included in the analysis. The final population in this study was 27,737 (Fig. 1).

### Basic examination

Data about lifestyle, such as smoking habits, physical activity, and antihypertensive medication were derived from the questionnaire. Smoking habits were categorized into three groups: smokers, ex-smokers, and never smokers. The diagnoses of diabetes and hypertension were based either on the subjects’ answers to the questionnaire about a doctor’s diagnosis or on the intake of medication for diabetes or hypertension. The diagnosis of PAD was based on questionnaire data, either a physician’s diagnosis, self-report, or previous peripheral arterial surgery.

Body weight was estimated using digital scales and the participants were lightly dressed without shoes. Height was measured to the nearest centimetre in the standing position with a fixed stadiometer. Body mass index (BMI) was calculated as weight (kg) divided by the square of the height (m^2^).

Blood lipids were analysed using a fasting venous blood sample, with standard methods at the local hospital laboratory.

Missing values were according to the following: hypertension medication = 901 (3.2%), hypertension = 887 (3.2%). All other variables had less than 1% missing values.

### Physical activity assessed by accelerometer

Accelerometry from a 7-day recording is a reliable assessment of habitual physical activity [14], but insensitive to some types of activity. A sensor-based tri-axial accelerometer, ActiGraph model wGT3X-BT (ActiGraph LCC, Pensacola, FL, USA) registered sedentary behavior and physical activity of the participants, who were instructed to wear the accelerometers for seven days in a belt around the right hip during waking hours, except during water-based physical activity. The software ActiLife v.6.13.3 was used to start the accelerometers and for the transfer and process of collected data. Accelerometer data expressing intensity of movements were displayed in counts per minute (cpm). Non-wear time was regarded as the time that the participants had no movements (0 cpm) for 60 or more consecutive minutes. Wear time was defined as 24 h minus non-wear time. Physical activity was expressed as percent of wake time spent in sedentary activities, moderate (MPA) and vigorous (VPA) physical activity by dividing time in each intensity with wear time.

Time spent in MPA and VPA was further categorized according to WHOs guidelines (low VPA < 75 min/week and low MPA < 150 min/week) [15]. As no limit for sedentary time is specified in the guidelines, high daily sedentary time was defined as 9.5 h/day. Accelerometer data were collected throughout the year, removing systematic bias of seasonal variability in physical activity and sedentary behavior [16].

Detailed information about the assessment of physical activity by accelerometer is described by Ekblom-Bak et al. [16].

### Blood pressure and ABI

Systolic and diastolic brachial blood pressures (SBP and DBP) were measured twice in both arms after 5 min rest in the supine position and the arm supported at heart level, using an Omron M10-IT blood pressure reader (Omron Corp, Kyoto, Japan). Blood pressures from the arm with highest mean SBP were used in the analysis. Mean systolic and diastolic brachial blood pressures were then calculated as the average of the two blood pressures registered.

ABI was also measured after at least 5 min of rest in the supine position, using Doppler measurements of brachial or radial systolic blood pressures as well as systolic blood pressures in arteria dorsalis pedis and tibialis posterior [17]. ABI for each ankle was measured as the highest of the pressures in each arteria dorsalis pedis or tibialis posterior over the average of the two supine brachial blood pressures in the arm with the highest blood pressures. Hence, one ABI value per ankle was obtained. Equipment which was used was Hadeco Bidop ES-100V3 (Hadeco Inc., Japan) or equivalent.

ABI was categorized as follows [17]:

1. ABI ≤ 0.9 in at least one leg: Low ABI.
2. ABI > 0.9 and < 1.0 in at least one leg: Borderline ABI.
3. ABI 1.0-1.39 in both legs: Normal ABI.
4. ABI ≥ 1.4 in at least one leg: High ABI.

### Statistics

The accelerometer-based percentages of sedentary time and moderate to vigorous physical activity, respectively, were divided into quartiles. ABI-categories were examined across the quartiles of physical activity and sedentary time.

One-way ANOVA was used to assess differences in cardiovascular risk factors between categories of physical activity or between the ABI-categories. Pearson’s chi2-test was used for dichotomous variables. A general linear model was used to adjust the relationships between physical activity measures and ABI categories for potential confounding factors. Model 1 was adjusted for age and sex. Model 2 also included adjustments for other established atherosclerotic risk factors (hypertension, diabetes, BMI, smoking, LDL) and factors that could influence registration of activity (the season of registration (four categories) and proportion of weekend days). To avoid overadjustment, we did not adjust for factors that could influence physical activity, without having any obvious causal relationship with atherosclerosis. Model 3 further included mutual adjustments for sedentary time and MVPA.

Tolerance was calculated for Model 3 to assess potential collinearity. Tolerance was > 0.4 for the four seasons, > 0.70 for sedentary time and MVPA, and > = 0.90 for all other covariates.

A sensitivity analysis was performed in which potential mediators (diabetes, hypertension, BMI) of the relationships between physical activity and ABI were excluded from the model.

We also combined high and low MVPA and time spent sedentary (SED) into four groups and used these as independent variables in a logistic regression using odds ratio (OR) to measure associations between physical activity and ABI. All tests were two-tailed with a significance level of 0.05.

IBM SPSS Statistics (v. 27, Armonk, NY, USA) software was used for all statistical calculations.

## Results

Characteristics of the study population in association with the accelerometer-based percentage of sedentary time are presented in Table 1. The most sedentary individuals (Q4) were mostly men, had higher brachial and ankle blood pressures, BMI, LDL, higher prevalence of type 2 diabetes, as well as higher percentage of low ABI, compared to the least sedentary group (Q1). However, there were no significant differences regarding mean ABI and prevalence of PAD between these two groups (Q4 and Q1).

**Table 1:** Baseline population characteristics and association with sedentary time assessed by accelerometer

Total 27,737	%sedentary time				
	Least sedentary			Most sedentary	p-value^*^
	Q1 (*n* = 7338)	Q2 (*n* = 7595)	Q3 (*n* = 6108)	Q4 (*n* = 6696)	
Sedentary time (%)	41	52	58	67	< 0.001
Age (years)	57.5 ± 4.3	57.5 ± 4.4	57.4 ± 4.4	57.6 ± 4.3	0.087
Women (%)	63.1	55.7	49.8	38.2	< 0.001
Systolic blood pressure (brachial) (mmHg)	124.7 ± 16.9	124.9 ± 16.6	126.1 ± 17.2	128.0 ± 17.2	< 0.001
Diastolic blood pressure (brachial) (mmHg)	76.5 ± 10.4	76.9 ± 10.4	77.7 ± 10.6	79.1 ± 10.6	< 0.001
Blood pressure right ankle (mmHg)	149.7 ± 19.7	150.2 ± 19.9	151.4 ± 20.6	153.1 ± 21.6	0.006
Blood pressure left ankle (mmHg)	148.7 ± 19.5	149.0 ± 19.9	150.2 ± 20.7	152.0 ± 21.4	< 0.001
Smoking (%)	13.3	11.7	11.0	12.3	< 0.001
Diabetes (%)	5.4	6.3	6.9	10.5	< 0.001
BMI	25.8 ± 4.0	26.5 ± 4.0	27.1 ± 4.3	28.3 ± 4.9	< 0.001
LDL (mmol/l)	3.4 ± 0.9	3.5 ± 0.9	3.5 ± 1.0	3.5 ± 1.0	< 0.001
Low ABI^**^ (%)	0.1	0.2	0.2	0.5	< 0.001
Hypertension (%)	19.2	21.2	22.8	27.4	< 0.001
Hypertension medication (%)	16.5	18.5	20.0	24.1	< 0.001
Peripheral artery disease (%)	0.3	0.2	0.3	0.4	0.218

^*^P-values calculated with 3 degrees of freedom

**ABI≤0.9

Characteristics of the study population in association with MVPA assessed by accelerometer are presented in Table 2. The least active (Q1) individuals were older, mostly women, smokers, had higher brachial blood pressure, BMI, LDL, higher prevalence of low ABI, diabetes, hypertension, PAD, and lower mean ABI compared to the most active ones (Q4). There were no differences between these two groups (Q1 and Q4) regarding mean systolic ankle blood pressures.

**Table 2:** Study population characteristics and association with moderate/vigorous physical activity assessed by accelerometer

Total 27,737	% moderate and vigorous activity time				
	least active			most active	*p*-value^*^
	Q1 (*n* = 8687)	Q2 (*n* = 7455)	Q3 (*n* = 5607)	Q4 (*n* = 5988)	
Moderate- and vigorous-intensity physical activity (%)	3	6	7	11	< 0.001
Age (years)	58.1 ± 4.3	57.4 ± 4.3	57.2 ± 4.3	57.1 ± 4.3	< 0.001
Women (%)	53.9	53.6	52.2	47.6	< 0.001
Systolic blood pressure (brachial, mmHg)	127.5 ± 17.5	125.7 ± 17.1	124.4 ± 16.4	125.0 ± 16.4	< 0.001
Diastolic blood pressure (brachial, mmHg)	78.6 ± 10.6	77.4 ± 10.7	76.7 ± 10.3	76.7 ± 10.2	< 0.001
Blood pressure right ankle (mmHg)	152.0 ± 21.3	151.0 ± 20.3	150.1 ± 19.8	151.1 ± 20.0	0.781
Blood pressure left ankle (mmHg)	150.6 ± 21.2	149.8 ± 20.3	148.9 ± 20.0	150.0 ± 19.7	0.685
Smoking (%)	16.9	11.6	9.2	8.7	< 0.001
Diabetes (%)	9.7	7.1	5.5	5.2	< 0.001
BMI	27.8 ± 4.8	26.8 ± 4.3	26.4 ± 4.1	26.1 ± 4.0	< 0.001
LDL (mmol/l)	3.5 ± 1.0	3.5 ± 1.0	3.4 ± 1.0	3.4 ± 1.0	< 0.001
Mean ABI^*^	1.2 ± 0.1	1.2 ± 0.1	1.2 ± 0.1	1.3 ± 0.1	< 0.001
Low ABI^**^ (%)	0.6	0.1	0.1	0.1	< 0.001
Hypertension (%)	27.6	21.9	19.2	19.2	< 0.001
Hypertension medication (%)	24.6	19.2	16.3	16.3	< 0.001
Peripheral artery disease (%)	0.5	0.2	0.2	0.2	0.002

* *P*−values calculated with 3 degrees of freedom

**Low ABI ≤0.9

Table 3 shows the association between ABI-categories and sedentary time and physical activity. Individuals with low-ABI were less active, more sedentary, had higher BMI, higher prevalence of diabetes, hypertension and smoking compared to those with normal ABI. Individuals with low-ABI had lower LDL, however, 23 individuals of 72 had medication for hyperlipidemia explaining the low LDL in this group.

**Table 3:** Association between ABI-categories and mean sedentary time and moderate vigorous physical activity percentage

	Low ABI ≤ 0.9	Borderline ABI > 0.9 and < 1	Normal ABI ≥ 1.0 and < 1.4	High ABI ≥ 1.4	*p*-value^*^
Number (total 27698)	72	298	24,963	2365	< 0.001
Mean age	60.0 ± 3.6	58.4 ± 4.3	57.5 ± 4.3	57.2 ± 4.3	< 0.001
Moderate to vigorous activity time %	0.03 ± 0.03	0.05 ± 0.03	0.06 ± 0.03	0.07 ± 0.04	< 0.001
Sedentary time %	0.60 ± 0.12	0.54 ± 0.11	0.54 ± 0.10	0.54 ± 0.10	< 0.001
BMI (kg/m^2^)	27.2 ± 4.7	28.0 ± 5.7	26.9 ± 4.4	26.9 ± 4.2	< 0.001
LDL(mmol/L)	3.2 ± 1.2	3.5 ± 1.1	3.5 ± 1.0	3.4 ± 1.0	< 0.001
Diabetes (%)	36.1	12.1	7.0	7.4	< 0.001
Systolic blood pressure (brachial, mmHg)	139.5 ± 19.1	134.4 ± 20.9	126.3 ± 16.9	120.1 ± 15.3	< 0.001
Diastolic blood pressure (brachial, mmHg)	79.7 ± 10.8	81.5 ± 12.0	77.8 ± 10.5	73.4 ± 9.8	< 0.001
Right ankle blood pressure (mmHg)	116.8 ± 26.9	136.5 ± 20.4	150.5 ± 19.8	159.2 ± 23.5	< 0.001
Left ankle systolic blood pressure (mmHg)	120.6 ± 26.0	135.9 ± 20.3	149.4 ± 19.8	157.3 ± 23.7	< 0.001
Smoking (%)	58.6	22.0	12.1	9.5	< 0.001
Hypertension (%)	56.5	28.3	22.7	19.1	< 0.001
Hypertension medication (%)	51.4	25.2	19.1	16.7	< 0.001

*P*-values calculated with 3 degrees of freedom

**Fig. 2:**
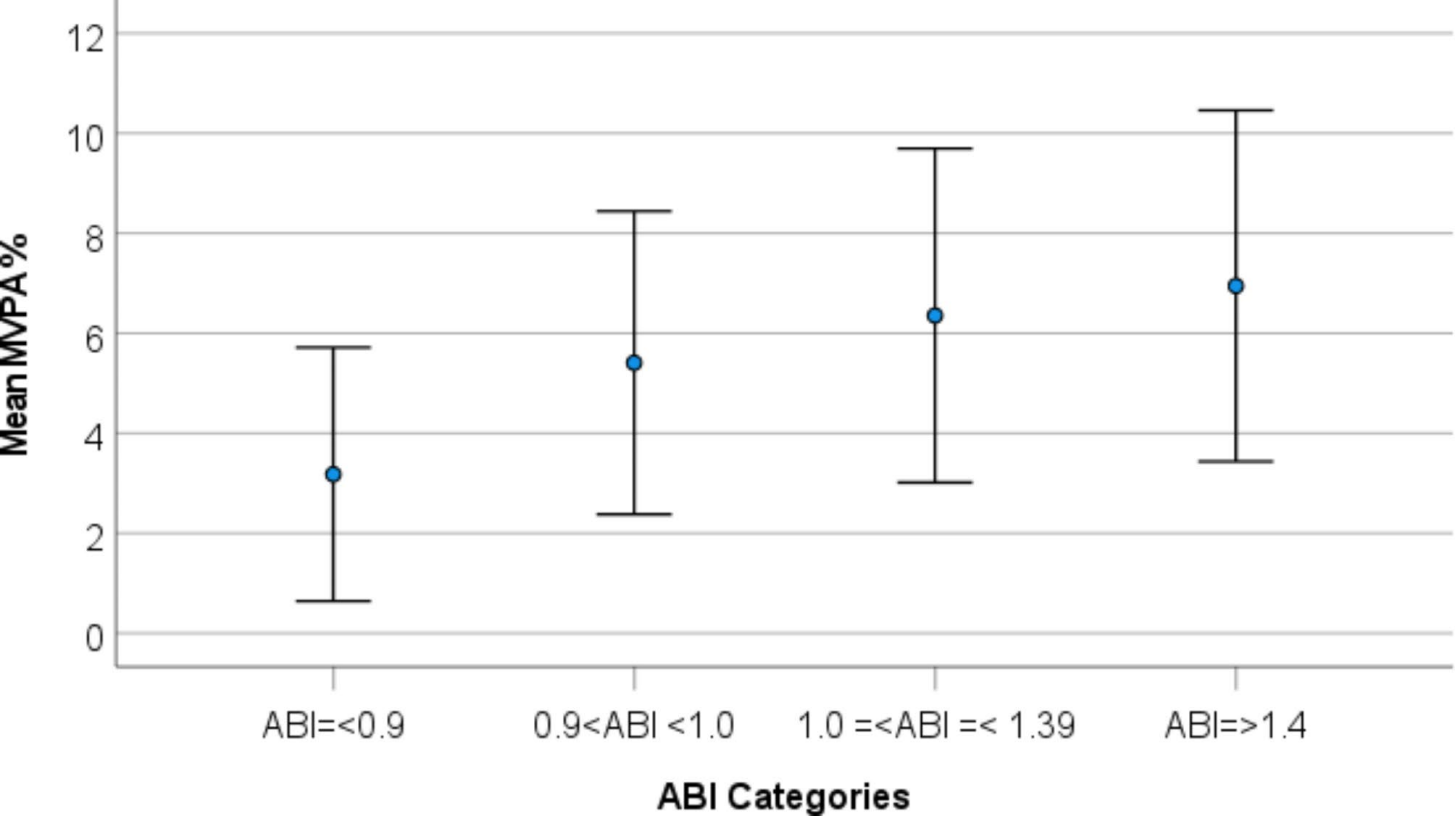
Association between mean MVPA% and ABI categories

**Fig. 3:**
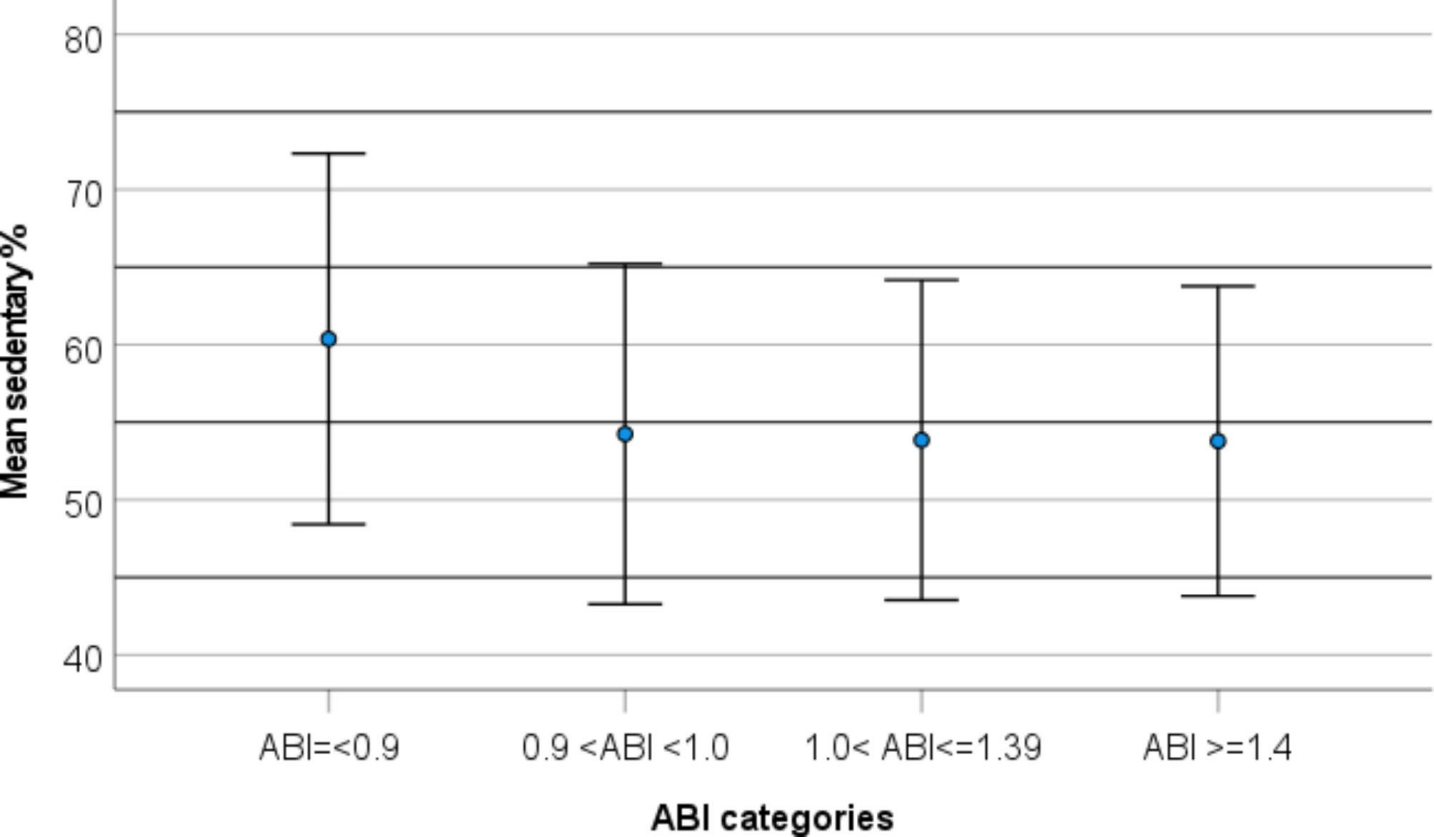
Association between mean sedentary% and ABI categories

Table 4 shows association between ABI-categories and MVPA and mean sedentary time in general linear regression models. As shown in the table, mean MVPA was lowest for low ABI in both models compared to other ABI-categories. Mean MVPA was highest for ABI > 1.4. Mean sedentary time was highest for low ABI and lowest for ABI > 1.4. These associations are also illustrated in Figs. 2 and 3. The results of model 3 indicate that MVPA was associated to high ABI, even when time spent sedentary was considered (p = < 0.001). However, time spent sedentary was not related to high ABI (*p* = 0.874). For low ABI, both sedentary time (*p* = 0.008) and time spent in MVPA (*p* < 0.001) were associated to the outcome.

**Table 4:** General linear regression models for ABI-categories and mean moderate/vigorous physical activity percent and mean sedentary percent, P-values denote difference vs. normal ABI (≥ 1 and < 1.4)

	Model 1^*^		Model 2^**^		Model 3^***^		Model 1^*^		Model 2^**^		Model 3^****^	
	Mean MVPA (95% CI)	*P* value	Mean MVPA (95% CI)	*P* value	Mean MVPA (95% CI)	*P* value	Mean Sedentary (95% CI)	*P* value	Mean Sedentary (95% CI)	*P* value	Mean Sedentary (95% CI)	*P* value
Low ABI ≤ 0.9	0.033 (0.025 to 0.040)	< 0.001	0.039 (0.031 to 0.047)	< 0.001	0.049 (0.042 to 0.056)	< 0.001	0.597 (0.574 to 0.621)	< 0.001	0.604 (0.580 to 0.628)	< 0.001	0.569 (0.548 to 0.591)	0.005
Borderline ABI > 0.9 and < 1	0.055 (0.051 to 0.059)	< 0.001	0.058 (0.055 to 0.062)	0.008	0.059 (0.056 to 0.063)	0.010	0.549 (0.537 to 0.560)	0.134	0.544 (0.533 to 0.556)	0.403	0.537 (0.526 to 0.547)	0.565
Normal ABI ≥ 1.0 and < 1.4	0.063 (0.063 to 0.064)	ref	0.064 (0.063 to 0.064)	ref	0.064 (0.063 to 0.064)	ref	0.540 (0.538 to 0.541)	ref	0.539 (0.538 to 0.541)	ref	0.539 (0.538 to 0.540)	Ref
High ABI ≥ 1.4	0.069 (0.067 to 0.070)	< 0.001	0.068 (0.067 to 0.070)	< 0.001	0.067 (0.066 to 0.069)	< 0.001	0.533 ± 0.002 (0.529 to 0-621)	0.001	0.533 (0.528 to 0.537)	0.002	0.539 (0.535 to 0.542)	0.983

*Adjusted for sex and age

**Adjusted for sex, age, diagnosed hypertension, diabetes, BMI, smoking, LDL, “season” and “weekend days percentage”

*** Adjusted for sex, age, diagnosed hypertension, diabetes, BMI, smoking, LDL, “season”, “weekend days percentage” and “mean sedentary percent”

**** Adjusted for sex, age, diagnosed hypertension, diabetes, BMI, smoking, LDL, “season”, “weekend days percentage” and “mean moderate/vigorous physical activity”

The results are presented as adjusted mean values (+ 95% CI). The adjusted mean values are calibrated to the mean of all covariates in the model. Number of participants in models are as following: model 1=27212, model 2 and model 3 = 26146

As diabetes, hypertension and BMI potentially could be mediators, and not only confounders, we excluded these variables from model 2 in a sensitivity analysis. The results did not change significantly. The analyses are presented as Supplementary Table 3.

When analysing the combined four categories of MVPA and SED, OR for low ABI was found to be significantly lower in the group with high MVPA- low SED, as well as in the group with high MVPA-high SED, compared to the reference (low MVPA-high SED). Low OR, but not significantly different from the reference group, was found for the low MVPA-low SED group (Supplement Fig. 1).

## Discussion

The present results showed that individuals with ABI > 1.4 had the highest mean MVPA. This group also had the lowest mean brachial blood pressure and the highest mean ankle blood pressure, which explains their high ABI. Time spent sedentary was negatively related to ABI. That indicates that time spent in MVPA was related to high ABI whereas times spent sedentary was related to lower ABI. MVPA was related to high ABI, even when time spent sedentary was considered, and seems to be more important than sedentary time for a high ABI. The results seem to be different regarding low ABI, i.e. both sedentary time and time in MVPA are related to the low ABI outcome. Hawkins et al. [11] found similar results for low ABI, but as individuals with high ABI were not included, the results of the present study add valuable information on this issue.

Traditionally, in a clinical setting, ABI > 1.4 has been considered as the result of non-compressible arteries, reflecting arterial stiffness, and suggested to be caused by arterial sclerosis or calcification. Diabetes, hypertension, metabolic syndrome, and old age amplify the vascular changes resulting in arterial stiffening [18]. In this study, however, individuals with ABI > 1.4 were not characterized by these risk factors. If anything, the risk factor profile was better in those with high ABI and the results remained significant even after adjustments for these risk factors.

The finding of higher mean MVPA in individuals with high ABI > 1.4 warrants further discussions both from an epidemiological but also physiological perspective. Of note, our current results are similar to those in the population-based ARIC study, which showed that individuals with a high ABI did not have a more adverse atherosclerosis risk factor profile and did not experience greater CVD event rates than those with a normal ABI during 12 years of follow-up [19]. This might indicate that high ABI in the general population is probably due to other physiological mechanisms than ABI > 1.4 in the clinical setting of PAD. Hoek et al. speculated that an elevated ABI is likely to be a multifactorial process in which, medial arterial calcification is not the only mechanism, but a variety of other factors play a role, such as exaggerated pulse pressure amplification which occurs in healthy individuals and is not associated with higher CVD risk [20]. A factor assumed to affect the ABI, that may explain our current findings, is the amount of lower extremity muscle mass, especially in muscular individuals [21]. According to the study by Tabara et al., in which muscle mass was measured by computed tomography and bioelectrical impedance, thigh muscle area, but not fat area, showed a strong positive association with ABI independent of BMI.

Similar to ARIC, there are a number of studies that question the assumption that high ABI should be interpreted as a proxy for PAD or medial arterial calcification [19, 20]. In subjects without CVD, the risk of cardiovascular events is not always higher in subjects with an abnormally high ABI than in subjects with a normal ABI [4, 22]. High ABI has even been associated with a lower risk of all-cause mortality compared to normal ABI, in another longitudinal study [22]. In the study by Jagt et al., high ABI was not associated with higher risk of either major adverse cardiovascular events or major adverse limb events among study subjects, whereas studies of patients with kidney disease have reported an increased risk of CVD in subjects with high ABI [23, 24].

The fact that studies show contradictory results when it comes to the association between high ABI and the risk of cardiovascular events might partially be due to differences between the study populations, e.g., if the study base is patients with high cardiovascular risk or individuals from the general population. One speculation might be that individuals with ABI > 1.4 is a very heterogenous group with both healthy individuals and those with PAD or other CVD risk. The positive association between high ABI and adverse CV outcomes in high-risk populations is likely related to medial arterial calcification being an etiologic factor [25]. These outcomes are quite consistent across studies. However, in studies reporting on general populations, the findings have been divergent [25]. Generally, studies investigating and analyzing subjects with ABI > 1.4 are rare. Since these individuals often have been excluded in different studies [26–29], our knowledge on this group is limited.

A study by Pellegrino et al. showed that football players had a lower ABI compared to runners and that vigorous strength training increases arterial stiffness [30]. This indicates that ABI and arterial stiffness might vary between athletes performing different sports. A randomized intervention study found a more pronounced arterial stiffness in strength-trained men compared to sedentary volunteers [31]. Hence, different physical activity modalities per se could have different physiological impact and thus different effects on arterial stiffness. More research is needed in this respect.

It is also possible that ABI could by falsely elevated in some individuals. According to Suominen et al., there are two reasons for falsely elevated ABI, either the use of a too narrow cuff or due to media sclerosis. In both these cases, clinical decision-making regarding PAD diagnosis becomes complicated [32].

Another result of our study and consistent with results from previous studies [10, 33], was that individuals with a higher percentage of time spent in MVPA had lower prevalence of low ABI, PAD, hypertension, and type 2 diabetes, as well as lower levels of BMI and LDL.

As both SED and MVPA were significantly related to ABI, we analyzed combinations of these measures. Results showed that in groups with high MVPA, the risk of low ABI were lower, irrespectively of the amount of SED. This may indicate that MVPA can alleviate the effect of high amounts of SED.

Based on the results of this study, higher percentage of time spent sedentary is positively associated with prevalence of low ABI, hypertension, and type 2 diabetes, as well as with higher BMI and LDL. This is in concordance with a recent study showing that having a low ABI is associated with lower physical activity [34]. Compared to the earlier studies [10, 33, 35], the SCAPIS population is much larger, but mean age is similar.

In two previous studies, a decline in physical activity among participants was mainly due to symptoms related to claudication [36, 37]. On the other hand, the study by Aldhahi et al. showed that a majority of participants with PAD, who were physically active, reported a lack of claudication symptoms (62%) [33]. It is unclear if the lower percentage of physical activity in our study is the consequence or cause of symptoms related to PAD.

As expected, individuals with low-ABI were less active, more sedentary, had higher BMI, higher prevalence of diabetes, hypertension, and smoking compared to those with normal ABI. This is in concordance with earlier studies showing association between low ABI and higher prevalence of diabetes, increased BMI and lack of exercise [38–41]. Due to the small number of individuals in the low ABI category in this study (72 out of 27,698), however, the results should be interpreted with caution.

### Strengths and limitations

One important strengths of this study is the large population-based sample with a final population of 27,737 individuals from the general population with information about ABI and physical activity assessed by accelerometer. Another strength is that the brachial blood pressure was measured both automatically and with doppler, and that there was no significant difference between these two measurements (supplementary Table 1). ABI calculation was based on doppler measurements. The assessment of physical activity and sedentary time by accelerometery over 1 week also strengthens the findings. Accelerometer assessed physical activity gives a more reliable estimate of actual daily activity than self-report methods, and as accelerometer data were collected during one week, throughout the year, seasonal bias was eliminated [16].

This study also has several limitations. The population was relatively healthy and young, aged 50–64 years old and, thus, not representing the usual age group with manifest PAD. Findings could thus not be translated to the groups which are usually targeted for interventions for PAD. As the study had cross-sectional design, we cannot assess causality. Further investigation, such as longitudinal studies to explore causality or studies to elucidate the underlying mechanisms, are needed to investigate temporal relationships. Ideally, such studies should include a broader age span than in our current study and investigate both healthy individuals and those with high risk for CVD.

Another study limitation was the lack of information about specific hypertensive treatment, which might have affected ABI or symptoms of PAD. Moreover, participants were instructed to wear the accelerometer only during waking time, but overnight wear might have occurred which would have increased the proportion of time spent sedentary and decreased the time spent physically active. The use of cut point (< 4 days) for determining valid days of accelerometery wear time has the potential to introduce bias into the study, as the less active or less healthy individuals may be less likely to achieve the threshold amount of wear time. Using ABI categories (instead of continuous data) might have led to some information loss, increasing the risk for type 2 error. ABI-categories have, however, been used in many other studies. Misclassification of exposure is potential cause of bias in most studies, and ABI could potentially have been misclassified in some individuals. Such error should be non-differential, however, and therefore bias the results towards null.

## Conclusion

This population-based study showed that middle-aged individuals with ABI > 1.4 had the highest mean level of physical activity according to accelerometry derived MVPA. As expected, individuals with lower ABI were less active and spent more time sedentary. Future studies are needed to better understand the associations between ABI, physical activity, and the risk of PAD and CVD in the general population.

### Electronic supplementary material

Below is the link to the electronic supplementary material.

Supplementary Material 1

## Data Availability

The data that support the findings of this study are available from SCAPIS but restrictions apply to the availability of these data, which were used under license for the current study, and so are not publicly available. Data are however available from the authors upon reasonable request and with permission of SCAPIS.

## Abbreviations

ABI: Ankle-Brachial Index
BMI: Body Mass Index
COPD: Chronic Obstructive Pulmonary Disease
CVD: Cardiovascular Disease
DBP: Diastolic Brachial Blood Pressures
LDL: Low Density Lipoprotein
MPA: Moderate Physical Activity
MVPA: Moderate to Vigorous Physical Activity
OR: Odds Ratio
PA: Physical Activity
PAD: Peripheral Artery Disease
SBP: Systolic Brachial Blood Pressures
SED: Sedentary
SCAPIS: Swedish CArdioPulmonary Bioimage Study
