# Supplementary material for: Associations between physical activity and ankle-brachial index: the Swedish CArdioPulmonary bioImage Study (SCAPIS)
Source: BMC Cardiovasc Disord. 2024 Aug 28;24:459. doi: 10.1186/s12872-024-04137-x (PMC11351556; doi:10.1186/s12872-024-04137-x)
Supplement: Supplementary file 1 — Supplementary Material 1 [file 12872_2024_4137_MOESM1_ESM.docx]

**Supplementary materials**
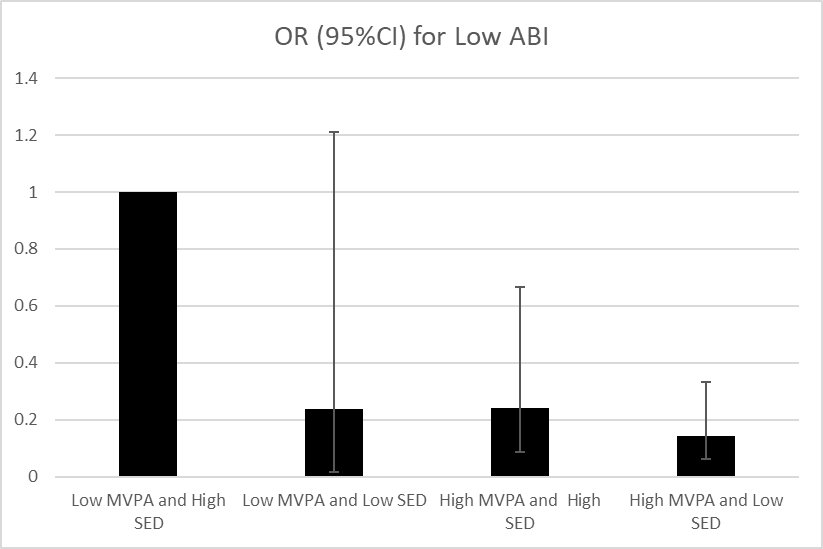


Supplement figure 1: Odds ratio (OR) for low ABI in relation to four categories of MVPA and time spent sedentary (SED). Analysed using logistic regression.

Supplementary table 1: Doppler and automatically measured systolic blood pressure (SBP).

|  | Number | Mean SBP | Standard deviation | Standard error Mean |
| --- | --- | --- | --- | --- |
| Doppler measured | 27733 | 125.4 | 17.2 | 0.1 |
| Automatically measured | 27733 | 125.9 | 17.0 | 0.1 |

Supplementary table 2. General linear regression models for ABI-categories and mean moderate/vigorous physical activity percent and mean sedentary percent, crude analysis.

|  | Crude value | | Crude value | |
| --- | --- | --- | --- | --- |
|  | Mean MVPA  ± Std Error | P value | Mean Sedentary  ± Std Error | P value |
| Low  ABI ≤ 0.9 | 0.032 ± 0.004 | <0.001 | 0.604 ± 0.012 | <0.001 |
| Borderline  ABI >0.9 and <1 | 0.054 ±0.002 | <0.001 | 0.543 ± 0.006 | 0.555 |
| Normal  ABI ≥1.0 and <1.4 | 0.063 ±0.001 | ref | 0.539 ± 0.001 | Ref |
| High  ABI ≥1.4 | 0.07 ±0.001 | <0.001 | 0.538 ± 0.002 | 0.589 |
|  |  |  |  |  |

Supplementary table 3: General linear regression models for ABI-categories and mean moderate/vigorous physical activity percent and mean sedentary percent

|  | Model 2 (Adjusted for sex, age, smoking, LDL, “season” and “weekend days percentage”) | |
| --- | --- | --- |
|  | Mean MVPA time (%)  (95% CI) | Mean Sedentary time (%)  (95% CI) |
| Low  ABI ≤ 0.9 | 0.037  (0.029 to 0.045) | 0.602  (0.578 to 0.626) |
| Borderline  ABI >0.9 and <1 | 0.056  (0.052 to  0.060) | 0.549  (0.538 to 0.561) |
| Normal  ABI ≥1.0 and <1.4 | 0.064  (0.063 to 0.064) | 0.539  (0.538 to 0.541) |
| High  ABI ≥1.4 | 0.069  (0.067 to 0.070) | 0.532  (0.528 to 0.536) |
